# Phylodynamic and Recombination Analyses of Avian Infectious Bronchitis GI-23 Reveal a Widespread Recombinant Cluster and New Among-Countries Linkages

**DOI:** 10.3390/ani11113182

**Published:** 2021-11-08

**Authors:** Mohamed H. Houta, Kareem E. Hassan, Matteo Legnardi, Claudia M. Tucciarone, Ahmed S. Abdel-Moneim, Mattia Cecchinato, Azza A. El-Sawah, Ahmed Ali, Giovanni Franzo

**Affiliations:** 1 Poultry Diseases Department, Faculty of Veterinary Medicine, Beni-Suef University, Beni-Suef 62511, Egypt; drhouta92@gmail.com (M.H.H.); kareem_eid@yahoo.com (K.E.H.); azzasawah@yahoo.com (A.A.E.-S.); 2 Department of Animal Medicine, Production and Health (MAPS), University of Padua, 35020 Legnaro, Italy; matteo.legnardi@phd.unipd.it (M.L.); claudiamaria.tucciarone@unipd.it (C.M.T.); mattia.cecchinato@unipd.it (M.C.); 3 Microbiology Department, Virology Division, College of Medicine, Taif University, Taif 21944, Saudi Arabia; asa@tu.edu.sa

**Keywords:** infectious bronchitis, IBV, GI-23, evolution, epidemiology, phylodynamic

## Abstract

**Simple Summary:**

Infectious bronchitis virus (IBV) is one of the main infectious agents affecting the avian industry. The remarkable evolutionary rate of this virus represents an often unsurmountable challenge to its control, leading to the emergence of different lineages featured by different biological properties and limited cross-protection. In the present study, the origin, spreading and evolution of GI-23, one of the most important IBV emerging lineages, has been reconstructed using a phylodynamic approach. To this purpose, the broadest available collection of complete and partial S1 sequences was downloaded from GenBank and merged with specifically sequenced European strains. After a likely ancient origin, GI-23 circulated undetected in the Middle East for a considerable time, thereafter emerging as a threat in parallel with the intensification of the poultry industry and its introduction in other countries. An intensive viral circulation affecting mainly neighbouring countries or those with strong economic and political relationships was demonstrated, even though some nations appear to play a major role as a “bridge” among less related locations. Of note, a big recombinant cluster, likely originating in the Middle East but spreading thereafter, especially to Europe through Turkey, demonstrated a much-marked increase in viral population size, and potentially fitness, compared to previously circulating variants.

**Abstract:**

Infectious bronchitis virus GI-23 lineage, although described approximately two decades ago in the Middle East, has recently drawn remarkable attention and is considered an “emerging” lineage due to its current spread to several other regions, including Europe. Despite the relevance, no comprehensive studies are available investigating its epidemiologic and evolutionary pattern. The present phylodynamic study was designed to fill this gap, benefitting from a collection of freely available GI-23 sequences and ad-hoc generated European ones. After a relatively ancient origin in the Middle East, likely in the first half of the previous century, GI-23 circulated largely undetected or underdiagnosed for a long time in this region, likely causing little damage, potentially because of low virulence coupled with limited development of avian industry in the considered years and regions and insufficient diagnostic activity. The following development of the poultry industry and spread to other countries led to a progressive but slow increase of viral population size between the late ‘90s and 2010. An increase in viral virulence could also be hypothesized. Of note, a big recombinant cluster, likely originating in the Middle East but spreading thereafter, especially to Europe through Turkey, demonstrated a much-marked increase in viral population size compared to previously circulating variants. The extensive available GI-23 sequence datasets allowed to demonstrate several potential epidemiological links among African, Asian, and European countries, not described for other IBV lineages. However, differently from previously investigated IBV lineages, its spread appears to primarily involve neighbouring countries and those with strong economic and political relationships. It could thus be speculated that frequent effective contacts among locations are necessary for efficient strain transmission. Some countries appear to play a major role as a “bridge” among less related locations, being Turkey the most relevant example. The role of vaccination in controlling the viral population was also tentatively evaluated. However, despite some evidence suggesting such an effect, the bias in sequence and data availability and the variability in the applied vaccination protocols prevent robust conclusions and warrant further investigations.

## 1. Introduction

Avian infectious bronchitis virus (IBV) is currently part of the species *Avian coronavirus*, genus *Gammacoronavirus,* family *Coronaviridae* order *Nidovirales* [1]. IBV genome is a single-stranded, non-segmented, positive-sense RNA approximately 27.6 kb long, with the following organization: 5′UTR-ORF1a/b-S-3a-3b-E-M-4b-4c-5a-5b-N-6b-3′UTR. The S gene encodes the spike protein, which is synthesized in an inactive form (S0) and subsequently cleaved by host proteolytic enzymes into S1 and S2 [2]. It is responsible for virus tropism, being involved in attachment and fusion with host cells [3] and represents the most important target of the host immune response. 

IBV mainly infects chickens [4], however, IBV and related viruses have also been detected in other avian species [1,5]. IBV infection is characterized by a short incubation period and primarily affects the respiratory tract. Thereafter, the virus can disseminate to various organs through viremia [6,7], and IBV tropism may vary depending on the strain involved [1]. 

Since its first identification in the 1930s, IBV has been reported worldwide, demonstrating a remarkable genetic variability. In fact, IBV is a rapidly evolving virus due to its high mutation (10^−3^–10^−5^ substitution/site/year) and recombination rates [2,8,9]. Such features have led to the emergence of several genetic variants featured by a remarkable genetic and phenotypic variability and often limited cross-protection [10,11].

Different classification systems have been used to track the evolution of IBV. The most widely recognized is based on the phylogenetic analysis of full S1 gene sequences [10]. According to this scheme, IBV is classified into eight genotypes, from GI to GVIII. In addition, each genotype is subclassified into genetic lineages [10,12,13,14,15]. 

The GI-23 is currently one of the most important and widespread lineages. Firstly detected in the Middle East [16,17] two decades ago, it then spread to other geographical regions, including Turkey (2010), Armenia (2012), Azerbaijan (2012), Lithuania (2012), Nigeria (2013), Kazakhstan (2015), Afghanistan (2016), Romania (2016), Poland (2016), Russia (2016), China (2018), and Germany (2019) as reviewed in Houta et al. [18]. 

Despite the relevance of this lineage and concerns among farmers and veterinarians, a formal analysis of its history, evolution, and spreading patterns has not been performed yet. The phylodynamic analysis, combining various statistical procedures, can be used to study how epidemiological, immunological, and evolutionary processes shape viral phylogenies [19], thus allowing the use of genetic data to estimate several population parameters. In the present study, a complete analysis of GI-23 was performed to investigate its epidemiology, evolutive and recombination patterns, population dynamics, and spreading process. The role of animal management, control strategies, and socio-economic factors was also considered and discussed to assess the potential impact on GI-23 population history.

## 2. Materials and Methods

### 2.1. European Dataset

Pools of tracheal samples (ten swabs/pool) were delivered from several European countries for routine diagnostic purposes to the laboratory of Veterinary Infectious Disease of the Dept. of Animal Medicine, Production and Health (Padua University, Padua, Italy). IBV diagnosis and preliminary characterization were performed based on the amplification and sequencing of the S1 hypervariable region 3 (HVR3) using the primer pair XCE1-2 [18]. Strains classified as GI-23 were confirmed and further characterized using in-house designed primer pairs allowing the amplification and sequencing of the whole S1 region in two overlapping PCRs (i.e., PCR1: S1_F 5′-GGAACGGTAAGGTGTTAGTT-3′ and S1Inner_R 5′-TTAGGCGAGGCATTACTTACTTAC-3′; PCR2: S1Inner_F 5′-GTGTGATAATTCACCAAGAGG-3′ and S1_R 5′-CATTCAGTAAAGGTGCCACAA-3′). Briefly, the following RT-PCR protocol was implemented using the SuperScript III Platinum One-Step RT-PCR Kit (Thermo Fisher, Waltham, MA, USA). Five µL of extracted RNA were added to a standard mix composed of 1 × reaction mix, 0.6 μM of PCR1 or PCR2 GI-23 specific primer pair, and 1 µL of SuperScript III RT/Platinum Taq Mix. Molecular-biology-grade water was added up to a final volume of 25 µL. The thermal protocol was 50 °C for 30 min, then 95 °C for 2 min, followed by 45 cycles of 95 °C for 15 s, 50 °C for 20 s, and 68 °C for 1 min. A final extension step at 68 °C for 5 min was performed. The positivity and specificity of the bands were verified by SYBR^TM^ Safe-stained (Thermo Fisher) agarose gel electrophoresis, and amplicons were Sanger-sequenced in both directions using the same PCR primers at Macrogen Spain (Madrid, Spain). Obtained sequences and related metadata were submitted to GenBank (Acc. Number MZ666004-MZ666086)

### 2.2. International Dataset

All IBV S1 sequences for which the collection date and country were available were downloaded from GenBank and aligned with the reference dataset provided by Valastro [10] using MAFFT [20]. A phylogenetic tree was reconstructed using IQ-Tree [21], selecting the substitution model with the lowest Akaike Information Criterion, calculated by the same program. Complete S1 sequences clustering in the GI-23 lineage were selected. Additionally, to improve the representativeness and geographic distribution resolution, the combined HVR1 and HVR2, and HVR3 sequences were also downloaded and classified using the same approach. Finally, the European and international datasets were merged and aligned using MAFFT. All sequences strongly clustering with vaccine strains were removed from the dataset.

### 2.3. Recombination Analysis

The resulting sequence collection was scanned for recombination occurrence using RDP4 [22]. The RDP4 settings for each method were adjusted accounting for the dataset features according to the RDP manual recommendations. Particularly, RDP, GENECONV, Chimaera, and 3Seq were used in the preliminary scan, while the full set of available methods was used for the analysis refinement. Only recombination events detected by more than two methods with a significance value lower than 10^−5^ (*p*-value < 10^−5^) and Bonferroni correction were accepted. Recombinant sequences were removed from the alignment, and recombination hot spots were also detected using the same program. Finally, the presence of residual recombination evidence and breakpoint locations were confirmed using GARD [23].

### 2.4. Phylodynamic Analysis

Non-recombinant datasets were analyzed using the Bayesian serial coalescent approach implemented in BEAST 1.8.2 [24] to jointly estimate several population parameters, including time to the most recent common ancestor (tMRCA), evolutionary rate, and viral population dynamics. Nucleotide substitution model (GTR+G) was selected based on the Bayesian information criterion (BIC) calculated using JModelTest [25], while the relaxed lognormal molecular clock and the Bayesian Skygrid were preferred over alternative models based on Bayesian factor (BF) evaluation, calculated through marginal likelihoods estimation through path sampling and stepping-stone methods, as suggested by Baele et al., [26]. A discrete state phylogeographic analysis was also performed as described by Lemey et al. [27]. The symmetric migration model was preferred over the asymmetric one based on BF, calculated as previously described. The Bayesian stochastic search variable selection (BSSVS) was also implemented, allowing to identify the most parsimonious description of the spreading process and calculate a BF indicative of the statistical significance of the inferred migration path between countries pairs [27]. For each dataset, three independent runs of 200 million generations were performed. The log and tree files were merged using *logcombiner* after removal of a burn-in of 20%. Results were analyzed using Tracer 1.6 and accepted only if the estimated sample size (ESS) was greater than 200 and the convergence and mixing were adequate. Parameter estimation was summarized in terms of mean and 95% highest posterior density (HPD). Maximum clade credibility (MCC) trees were constructed and annotated using TreeAnnotator (BEAST package). SpreaD3 was used to display the spreading process over time and to calculate the BF associated with each migration route [28]. All non-zero transition rates among countries were considered significant when the BF was greater than 10. Additional summary statistics were calculated for the different runs using homemade specific R scripts.

## 3. Results

### 3.1. Dataset

A total of 231 complete S1 sequences, 270 HVR1/2, and 341 HVR3 were included in the initial dataset, representing 8, 12, and 14 countries, respectively (Table 1 and Supplementary data 1). Differently from a previous systematic review article using some sequences of GI-23 for intragenotype phylogenetic analysis [20], the present study uses all possible resources (complete and partial sequences) to reconstruct the evolutionary dynamics of GI-23 over the time and space since its first emergence.

### 3.2. Recombination Analysis

**Figure 1:**
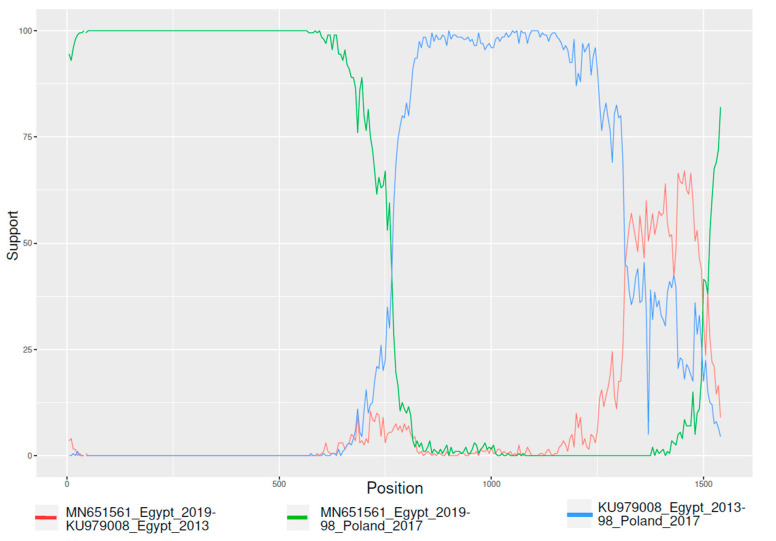
Result of bootscan analysis performed with RDP4. For reasons of clarity, only one sequence representative of recombinant, major, and minor parents are reported. Y and x-axis depict the bootstrap support and the nucleotide position of the S1 gene.

The complete S1 sequences dataset demonstrated significant evidence of recombination, and a breakpoint at approximately position 780 of the S1 alignment was consistently detected by RDP4 and GARD (Figure 1).

Therefore, the alignment was split in the two regions based on detected recombination breakpoint, which were further tested for recombination occurrence. Since no evidence of residual recombination signal was detected, they were analyzed in independent runs. Accordingly, the combined HVR1, HVR2, and HVR3 displayed no recombination evidence in the considered region.

Notably, a numerous recombinant cluster was detected and included mainly, but not only, European sequences. Both major and minor parental strains were part of clusters composed mainly of Egyptian GI-23 strains. Because of the potential epidemiological relevance of this cluster, its history was independently estimated after excluding the presence of within-cluster recombination. Overall, the following datasets were created and analyzed: (a) N-terminal of the S1 region (based on strains for which a complete S1 sequence was available), (b) C-terminal of the S1 region (based on strains whose complete S1 sequence was available), (c) Recombinant cluster, d) HVR1/2, and e) HVR3.

Finally, it was noted that strains Israel720/99 (AY091552) and IS/885 (AY279533), sampled in Israel in and 1999 and 2000, although classified as GI-23 in Valastro et al., showed a genetic distance compared to other reference strains higher than 15%. Such percentage is higher than the cut-off defined by the proposed classification itself; therefore, they were initially excluded from the analysis. However, for comparison purposes, two additional analyses (A.1 and B.1) of N-terminal and C-terminal of the S1 region were performed, including these strains. The inclusion of the highly divergent GI-23 sequences (i.e., dataset A.1 and B.1), despite causing the estimation of a more ancient tMRCA, did not significantly affect the overall lineage population dynamics and spreading patterns (data not shown). Therefore, the ambiguity in GI-23 sequence classification can be considered of negligible practical relevance and was not further considered.

### 3.3. Phylodynamic Analysis

**Figure 2:**
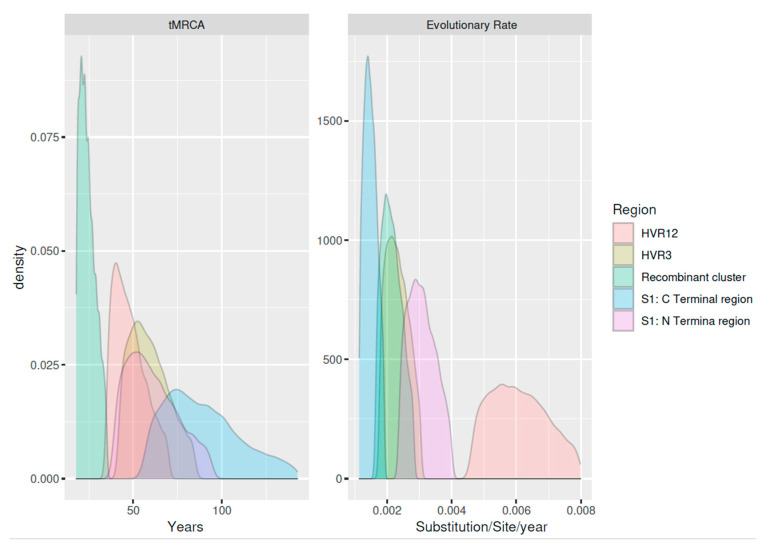
Density plots reporting the estimated tMRCA (**left**) and evolutionary rate (**right**). Results of different datasets have been color-coded.

Estimates of the main population parameters and a summary of the dataset features are reported in Table 1 and Figure 2. 

**Figure 3:**
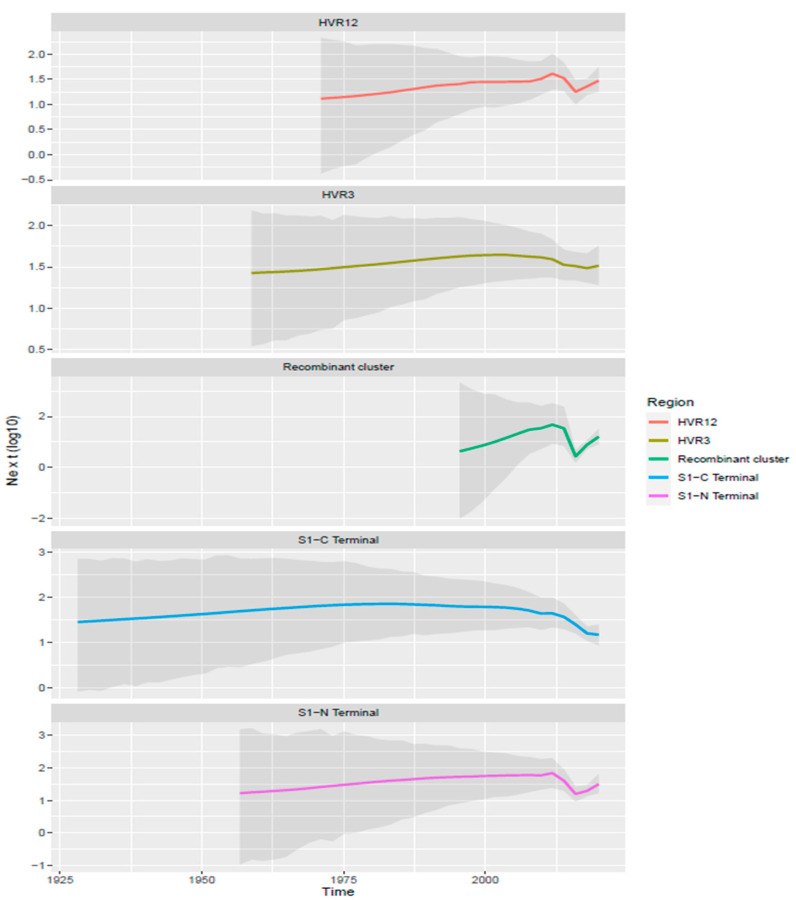
Skygrid reconstruction of GI-23 population dynamics over time. The mean value of relative genetic diversity (Ne∙t) is depicted by a continuous line, while shaded areas represent the 95HPD intervals. Different datasets have been color-coded and facetted.

The evaluation of the viral population dynamics demonstrated a consistent pattern between datasets A and D, and B and E (Figure 3). Overall, a persistent low-level circulation, slowly but constantly increasing, was observed until approximately 2006–2010. Afterward, a decrease in the relative genetic diversity featured HVR3 and C-terminal datasets, while a second increase from about 2015 could be observed in the HVR1/2 and N-terminal regions. A partially overlapping scenario featured the recombinant cluster that, after its estimated origin in the middle of the 1990s, showed a rapid expansion, followed by a marked decline and rebound in the period between 2010 and 2020.

**Figure 4:**
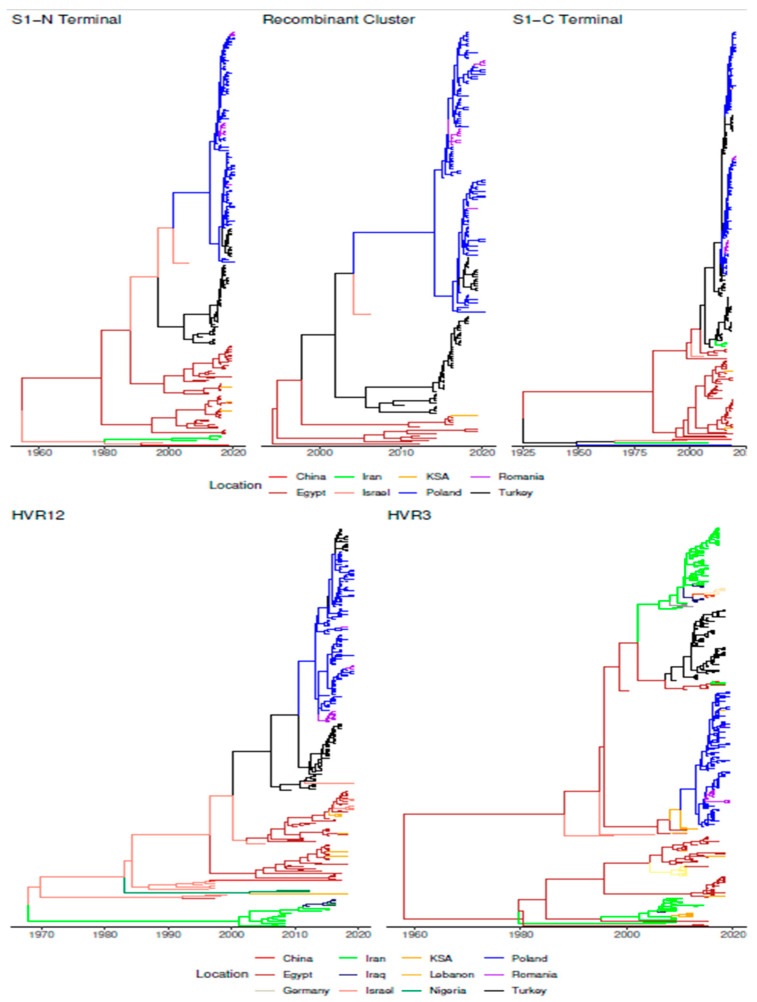
Time calibrated phylogenetic trees obtained for the different datasets runs are reported. The tree branches have been colour-coded according to the location predicted with the highest posterior probability.

**Figure 5:**
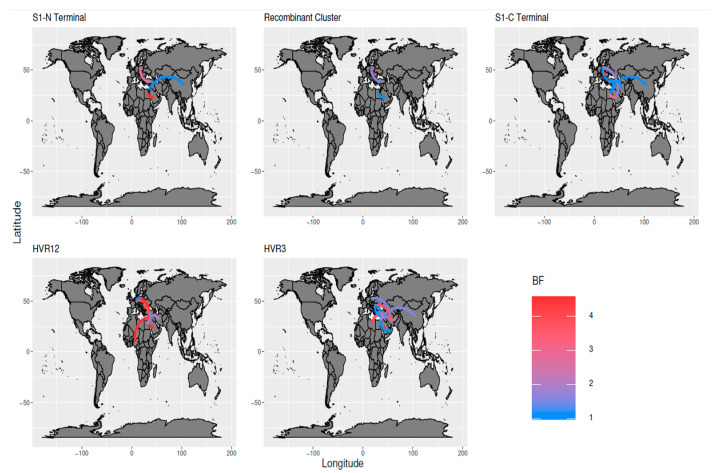
Well supported migration paths among countries are depicted as edges whose colour is proportional to the BF of the migration rate. The location of each country has been matched with its centroid.

Phylogeographic analysis suggested a possible lineage origin in the Middle East at least in the 1950s, most likely in Palestine, the area currently corresponding to Palestine/Israel or, with a lower probability, in Egypt. Thereafter, GI-23 spread to other Middle East countries, including the Kingdom of Saudi Arabia (KSA) and Turkey, which represented the most likely source of viral introduction to Europe (Posterior Probability (PP) = 1). Israel was inferred as the potential source of viral introduction to China (PP = 0.85), although Iranian strains were part of the same cluster (Figure 4 and Figure 5).

Particularly, the recombinant cluster (dataset C) appears to have followed this “European path” differently from other strains which circulated mainly in the Middle East. After a likely Egyptian origin, where most of the strains of both minor and major parent clusters were sampled, the new recombinant strain spread to Turkey (~2000) and after that to Europe (~2010–2015), most likely in Poland. The following entry in Romania, through multiple introduction events, has also been demonstrated with strong statistical support (PP > 0.99). A reintroduction to Turkey also appears to have occurred. 

The analysis of all sequences, from which HVR1, HVR2, or HVR3 region was available, confirmed a similar pattern, although with a higher spatial resolution and expanding the available area. A Middle East (Egyptian or Israeli) origin was predicted from both genomic regions, likely depending on the differential sequence availability. Combining the results of HVR1/HVR2 and HVR3 region analysis revealed that after an introduction from Egypt, Iran played a major role in further GI-23 spreading in the Middle East countries, like Kurdistan and Iraq (PP = 0.97), which in turn was involved in the spread to Afghanistan (PP = 0.95) and Syria (PP = 0.93). Based on the HVR3 dataset, Iran was also predicted as a potential source for China (PP = 0.83). In parallel, Egypt was involved in the viral introduction to Libya (PP > 0.90) and Oman (PP > 0.55) through multiple introduction events, while strains detected in Lebanon (PP = 0.99) and Nigeria (PP = 0.88) presented a most likely introduction from Israel (Figure 4 and Figure S1).

Interestingly, the HVR1/HVR2 and HVR3 of the strains clustering into the recombinant group demonstrated the same spatial distribution as the one identified using the whole S1 dataset, except for a recombinant strain detected in Lebanon (HVR1/HVR2) and a few in Libya, Oman, and Kurdistan (HVR3) (Figure 4). Statistically supported migration rates (BF > 10) highlighted the consistency of this pattern, with well-supported migration, especially within Europe, the Middle East, and the Far East. Turkey and Iran appeared to be the main “bridges” mediating inter-area viral dispersal (Figure 5).

## 4. Discussion

IBV has been demonstrated to be a virus with a remarkable evolutionary potential, which has led to the emergence of several genotypes and lineages, some of those featured by outstanding epidemiological success. Among these lineages, the GI-23 was initially discovered in Middle East countries and has been more recently identified out of this area and reported in the Far East, Africa, and Europe [17,18,29,30,31,32]. 

Unfortunately, coherent and standardized sampling and sequencing protocols are still not consistently adopted. The remarkable heterogenicity in sequence availability, especially the sequencing of different, non-overlapping regions, can lead to poorly comparable or even conflicting results. Furthermore, different genomic regions of strains evolving in different countries and/or geographic regions can have a different evolutive history. Despite the implemented phylodynamic approach being more robust to unbalanced and incomplete information than classical epidemiology, the varying effort put in by different countries into monitoring and sequencing activities might also affect the inference of GI-23 history, with a particular effect on its spreading over time, and some among-countries linkages could have been missed. For this reason, we created several datasets to explore the whole available information pool. Independently of the considered dataset, the present study results confirm some features previously demonstrated for other IBV lineages like GI-16 and GI-19, i.e., the high evolutionary rate and the ancient origin and prolonged undetected circulation [8,33]. Strains belonging to this genotype showed a comparable evolution rate with other ssRNA viruses, coronaviruses and other IBV variants representing a huge menace for animal health and farm profitability [8,33,34]. Thus, GI-23 can also be inferred to have a similar potential. The combined evaluation of viral population dynamics and migration patterns can help to understand the epidemiology of GI-23 after its origin and complex evolution, which needed several co-factors contributing to the successful emergence of new variants. 

GI-23 appears to have circulated for a long time in the Middle East countries, likely causing limited damages [16,17,35,36,37,38,39]. This evidence could be ascribed to different factors: (1) an initially low viral virulence, potentially followed by progressive evolution that has led to a better adaptation, infectiveness, virulence, etc.; (2) a limited development of avian industry in the considered years and regions, (3) an insufficient diagnostic activity, leading to underreporting, even if the coalescent-based analysis should at least partially compensate for this bias.

Thereafter, between the late 1990s and 2010, a progressive but slow increase of the viral population size occurred, which likely reflected the spread to other countries combined with an alteration in the abovementioned “risk” factors. Noteworthy, several sequenced strains and predicted lineages were of Israeli and Turkish origin within the same time frame. The advanced poultry industry in both countries created favourable environmental conditions for viral expansion; meanwhile, better veterinary services and diagnostic tools to detect the viruses were available [1,2,40,41]. Potentially, the late development of poultry production in some Middle East regions could have delayed the expansion of GI-23 lineage compared to others like the QX (GI-19) and Q1 (GI-16) that had an earlier circulation in highly developed countries [8,33].

The following “decrease and rebound” in viral effective population size observed after 2010 is more challenging to explain. A likely explanation could involve the “masking” by high pathogenicity avian influenza (HPAI), which attracted most of diagnostic and research efforts and pushed for higher biosecurity measures [42]. Interestingly, a homologous vaccine was developed and applied in some Middle East countries, in Egypt, Israel and thereafter in Turkey and Kurdistan ([43,44,45,46,47,48] and personal communication). The limited poultry population involved in vaccination protocols could hardly explain the remarkable observed effect. However, it must be stressed that a relevant percentage of available sequence data originated from these regions. Therefore, the observed decline could reflect a local pattern due to intense vaccine application rather than a global pattern, where the overall impact would have been limited. Nevertheless, other hypotheses and epidemiological factors could justify the observed scenario and would be worthy of investigation when more sequences and data would become available. 

The present evidence testifies the GI-23 circulation in few European countries [29,30,47], and its spread appears much less effective compared to the one reported for other lineages after their introduction in Europe [8,33]. Similarly, even in the Middle East and Asia, viral expansion to new countries seems to involve primary neighbouring countries and those with strong economic and political relationships. It could thus be speculated that frequent effective contact among countries is necessary for efficient strain transmission. 

The viral evolution must be considered, hence our study also reports the emergence and spread of a major recombinant clade since the late 1990s, likely originating in Egypt. It must be stressed that recombination analysis is one of the most challenging tasks of viral phylogenetics. Despite the efforts, the current methods are still perfectible. The limited distance between parental strains and/or the progressive evolution after recombination that modify the original recombinant sequence can definitely complicate the detection of ancestral recombination events. Consequently, the confident identification of actual recombinant and parental strains is probably far from being achieved. Nevertheless, the use of conservative analysis parameters, the consistency among different methods, and the phylogenetic evidence support the robustness of our results.

This recombinant clade had a sharper expansion than other GI-23 populations even during its circulation in the Middle East regions [48], supporting a higher virulence/fitness than parental strains. A decline followed the intense rise, probably due to effective control measures implementation and crescent population immunity. A second major peak was observed after GI-23 viruses introduction to Europe, where a naïve host population was present, and the implementation of specific vaccination strategies was limited and tardive, or even totally absent [30,49]. Additionally, more intense diagnostic sequencing activity in European countries likely contributed to an overrepresentation of available sequences. 

Interestingly, European poultry populations are mainly vaccinated with a combination of Mass plus 793B or Mass plus QX vaccines to protect against the highly prevalent QX field strains [49]. Although cross-protection was demonstrated experimentally, this issue deserves to be further investigated as it is unclear whether these vaccinations display a limited efficacy against GI-23 in field conditions or if a difference exists between the protocols that could justify the expansion of GI-23 in Europe [45,49]. 

Remarkably, some of the highlighted migration links were already inferred for the QX genotype, like the within Europe contacts or between Iran and Iraq [3,4,5,8,50,51]. However, the present study allowed to formally investigate and statistically assess several additional connections among regions, like northern Africa, or low-income countries of Asia, that had been largely neglected. Expectedly, viral migration appears to be highly dependent on the relationships among countries, and therefore by cultural, historical, and political barriers, which can noticeably affect viral dispersal. Bridge countries among these locations were also identified, being Turkey the most relevant example. Migratory birds should also be taken into consideration according to the recent report of GI-23 in wild and migratory birds in Egypt [52]. Although the overall migration patterns and features appear clear and consistent among different datasets, some incongruences were present. It cannot be underemphasized that the occurrence, rate and timing of specific connections between country pairs should be always treated with caution due to the unavoidably biased nature of the available sequences, particularly those collected far in the past. Even though efforts were paid to maximize the available information, most of the circulating strains were not sampled or sequenced, and the effect of chance in this process had surely affected some of the analysis results. A more intense sampling activity, while desirable, was impossible due to the limited resources of the countries where the virus initially circulated. Moreover, sequencing was overall rarely performed decades ago and the retrospective nature of the study hinders any effective remedy. Nevertheless, rather than weakening the present study results, this scenario emphasizes the utility and power of statistical and modelling approaches, which were repeatedly proven capable of effectively deal with such data shortage, and it acts as a warning on the pivotal relevance of intense and balanced sequencing activity, especially in light of remarkable advances in the field. However, it must also be considered that sampling and sequencing activities, although less intense than in present days, were already underway in Africa, Asia, Middle East and especially in Europe well before the first GI-23 strain identification, either in the considered countries and/or in nearby ones. Therefore, the evidence that other Variant2 strains were not reported before and/or in other countries supports, with a certain confidence, that this genotype was not circulating. The European example is particularly obvious since thousands of sequences were obtained for decades before the first detection of Varaint2 strains. Therefore, this evidences represents a point in favour of our sampling reliability.

## 5. Conclusions

Taken together, the GI-23 lineage history can be considered a compelling depiction of the “epidemiological triad” concept, being the interaction of viral (i.e., a potentially more virulent recombinant cluster), host (i.e., more extensive, denser, stressed animal population of selected genetic lineages) and environmental (i.e., farming conditions and management, co-infections, control strategies, etc.) factors involved in the determination of the virus epidemiology. A comparable scenario, partially dictated by deterministic factors and partially by stochastic/random ones, could explain the differential success of several IBV lineages detected all around the world over time. The study of different viral variants can thus allow exploring previously unreported epidemiological linkages and the underlying factors determining them. Therefore, further studies should be encouraged to increase the overall knowledge on the topic, which could likely be extended to other infectious diseases of veterinary and human interest. In this sense, the herein reported approach could find immediate application as a model for future studies dealing with poorly known IBV genotypes and/or other geographic areas. Finally, the encountered limitations due to the sparse and biased sequence availability should act as a warning on the relevance of implement a routine, intense and balanced sequencing activity for major infections of veterinary and human interest, especially in light of the remarkable advances in sequencing technologies and their accessibility.

## Figures and Tables

**Table 1:** Summary of the main dataset features and estimates of the population parameters reported as mean, 95% lower, and higher posterior density (95HPD).

					Time to the Most Recent Common Ancestor (Years)			Origin			Evolutionary Rate		
Database	Included Sequences	Sequence Number	Years	Country Number	Mean	Lower 95HPD	Higher 95HPD	Mean	Lower 95HPD	Higher 95HPD	Mean	Lower 95HPD	Higher 95HPD
**A**	S1-N terminal	231	1998–2020	8	58.737	43.828	81.972	1961.263	1938.028	1976.172	3.05 × 10^−3^	2.54 × 10^−3^	3.69 × 10^−3^
**B**	S1-C terminal	231	1998–2020	8	86.130	64.250	120.229	1933.87	1899.771	1955.75	1.48 × 10^−3^	1.23 × 10^−3^	1.78 × 10^−3^
**C**	Recombinant cluster	188	2006–2020	6	23.618	19.008	31.081	1996.382	1988.919	2000.992	1.77 × 10^−3^	2.13 × 10^−3^	2.60 × 10^−3^
**D**	HVR1/2	270	1996–2020	12	57.942	45.787	75.2	1962.058	1944.8	1974.213	6.02 × 10^−3^	4.96 × 10^−3^	7.33 × 10^−3^
**E**	HVR3	341	1998–2020	14	46.622	37.628	61.372	1973.378	1958.628	1982.372	1.90 × 10^−3^	2.30 × 10^−3^	2.81 × 10^−3^

## Data Availability

All the sequences obtained in the present study have been made freely available in GenBank and the relative accession numbers are reported in supplementary data 1.

## Supplementary Materials

The following are available online at https://www.mdpi.com/article/10.3390/ani11113182/s1, Supplementary Figure S1. Time calibrated phylogenetic trees based on the A, B, C, D and E dataset are represented in each page. The tree branches have been color-coded according to the location predicted with the highest posterior probability. Supplementary data 1. List of sequences and relative metadata used in the present study. For each sequence the collection country and year are reported. Sequences have been divided according to the available region and the dataset (i.e., European or internationa)
