# Supplementary material for: Phylodynamic and Recombination Analyses of Avian Infectious Bronchitis GI-23 Reveal a Widespread Recombinant Cluster and New Among-Countries Linkages
Source: Animals (Basel). 2021 Nov 8;11(11):3182. doi: 10.3390/ani11113182 (PMC8614413; doi:10.3390/ani11113182)
Supplement: Supplementary file 1 [file animals-11-03182-s001.zip › Figure S1.pdf]

Supplementary figure 1. Time calibrated phylogenetic trees based on the A, B, C, D and E dataset are represented in each page. The tree branches have been color-coded according to the location predicted with the highest posterior probability.

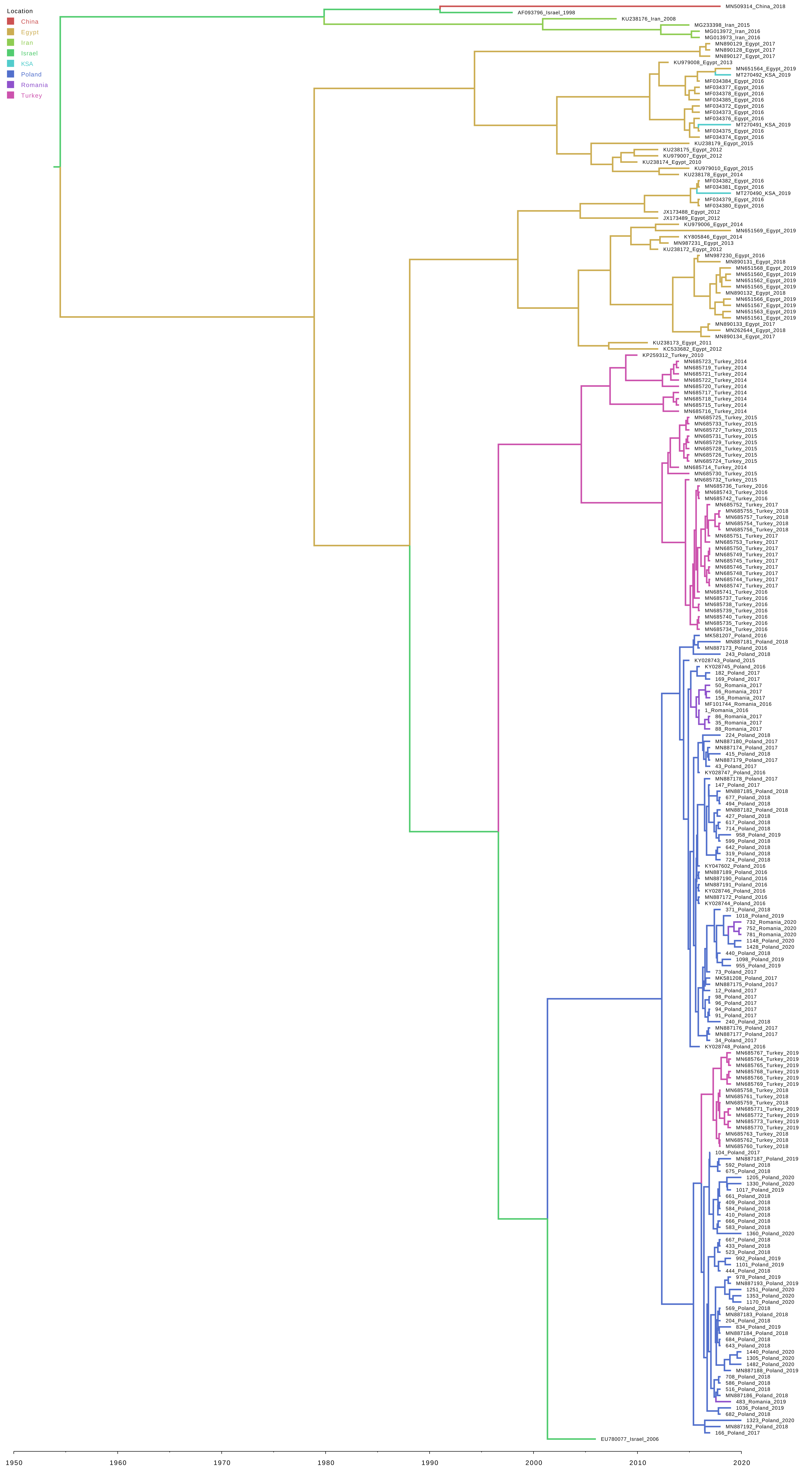
Location
China
Egypt
Iran
Israel
KSA
Poland
Romania
Turkey
MN509314_China_2018
AF093796_Israel_1998
KU238176_Iran_2008
MG233398_Iran_2015
MG013972_Iran_2016
MG013973_Iran_2016
MN890129_Egypt_2017
MN890128_Egypt_2017
MN890127_Egypt_2017
KU979008_Egypt_2013
MN651564_Egypt_2019
MT270492_KSA_2019
MF034384_Egypt_2016
MF034377_Egypt_2016
MF034378_Egypt_2016
MF034385_Egypt_2016
MF034372_Egypt_2016
MF034373_Egypt_2016
MF034376_Egypt_2016
MT270491_KSA_2019
MF034375_Egypt_2016
MF034374_Egypt_2016
KU238179_Egypt_2015
KU238175_Egypt_2012
KU979007_Egypt_2012
KU238174_Egypt_2010
KU979010_Egypt_2015
KU238178_Egypt_2014
MF034382_Egypt_2016
MF034381_Egypt_2016
MT270490_KSA_2019
MF034379_Egypt_2016
MF034380_Egypt_2016
JX173488_Egypt_2012
JX173489_Egypt_2012
KU979006_Egypt_2014
MN651569_Egypt_2019
KY805846_Egypt_2014
MN987231_Egypt_2013
KU238172_Egypt_2012
MN987230_Egypt_2016
MN890131_Egypt_2018
MN651568_Egypt_2019
MN651560_Egypt_2019
MN651562_Egypt_2019
MN651565_Egypt_2019
MN890132_Egypt_2018
MN651566_Egypt_2019
MN651567_Egypt_2019
MN651563_Egypt_2019
MN651561_Egypt_2019
MN890133_Egypt_2017
MN262644_Egypt_2018
MN890134_Egypt_2017
KU238173_Egypt_2011
KC533682_Egypt_2012
KP259312_Turkey_2010
MN685723_Turkey_2014
MN685719_Turkey_2014
MN685721_Turkey_2014
MN685722_Turkey_2014
MN685720_Turkey_2014
MN685717_Turkey_2014
MN685718_Turkey_2014
MN685715_Turkey_2014
MN685716_Turkey_2014
MN685725_Turkey_2015
MN685733_Turkey_2015
MN685727_Turkey_2015
MN685731_Turkey_2015
MN685729_Turkey_2015
MN685728_Turkey_2015
MN685726_Turkey_2015
MN685724_Turkey_2015
MN685714_Turkey_2014
MN685730_Turkey_2015
MN685732_Turkey_2015
MN685736_Turkey_2016
MN685743_Turkey_2016
MN685742_Turkey_2016
MN685752_Turkey_2017
MN685755_Turkey_2018
MN685757_Turkey_2018
MN685754_Turkey_2018
MN685756_Turkey_2018
MN685751_Turkey_2017
MN685753_Turkey_2017
MN685750_Turkey_2017
MN685749_Turkey_2017
MN685745_Turkey_2017
MN685746_Turkey_2017
MN685748_Turkey_2017
MN685744_Turkey_2017
MN685747_Turkey_2017
MN685741_Turkey_2016
MN685737_Turkey_2016
MN685738_Turkey_2016
MN685739_Turkey_2016
MN685740_Turkey_2016
MN685735_Turkey_2016
MN685734_Turkey_2016
MK581207_Poland_2016
MN887181_Poland_2018
MN887173_Poland_2016
243_Poland_2018
KY028743_Poland_2015
KY028745_Poland_2016
182_Poland_2017
169_Poland_2017
50_Romania_2017
66_Romania_2017
156_Romania_2017
MF101744_Romania_2016
1_Romania_2016
86_Romania_2017
35_Romania_2017
88_Romania_2017
224_Poland_2018
MN887180_Poland_2017
MN887174_Poland_2017
415_Poland_2018
MN887179_Poland_2017
43_Poland_2017
KY028747_Poland_2016
MN887178_Poland_2017
147_Poland_2017
MN887185_Poland_2018
677_Poland_2018
494_Poland_2018
MN887182_Poland_2018
427_Poland_2018
617_Poland_2018
714_Poland_2018
958_Poland_2019
599_Poland_2018
642_Poland_2018
319_Poland_2018
724_Poland_2018
KY047602_Poland_2016
MN887189_Poland_2016
MN887190_Poland_2016
MN887191_Poland_2016
KY028746_Poland_2016
MN887172_Poland_2016
KY028744_Poland_2016
371_Poland_2018
1018_Poland_2019
732_Romania_2020
752_Romania_2020
781_Romania_2020
1148_Poland_2020
1428_Poland_2020
440_Poland_2018
1098_Poland_2019
955_Poland_2019
73_Poland_2017
MK581208_Poland_2017
MN887175_Poland_2017
12_Poland_2017
98_Poland_2017
96_Poland_2017
94_Poland_2017
91_Poland_2017
240_Poland_2018
MN887176_Poland_2017
MN887177_Poland_2017
34_Poland_2017
KY028748_Poland_2016
MN685767_Turkey_2019
MN685764_Turkey_2019
MN685765_Turkey_2019
MN685768_Turkey_2019
MN685766_Turkey_2019
MN685769_Turkey_2019
MN685758_Turkey_2018
MN685761_Turkey_2018
MN685759_Turkey_2018
MN685771_Turkey_2019
MN685772_Turkey_2019
MN685773_Turkey_2019
MN685770_Turkey_2019
MN685763_Turkey_2018
MN685762_Turkey_2018
MN685760_Turkey_2018
104_Poland_2017
MN887187_Poland_2019
592_Poland_2018
675_Poland_2018
1205_Poland_2020
1330_Poland_2020
1017_Poland_2019
661_Poland_2018
409_Poland_2018
584_Poland_2018
410_Poland_2018
666_Poland_2018
583_Poland_2018
1360_Poland_2020
667_Poland_2018
433_Poland_2018
523_Poland_2018
992_Poland_2019
1101_Poland_2019
444_Poland_2018
978_Poland_2019
MN887193_Poland_2019
1251_Poland_2020
1353_Poland_2020
1170_Poland_2020
569_Poland_2018
MN887183_Poland_2018
204_Poland_2018
834_Poland_2019
MN887184_Poland_2018
684_Poland_2018
643_Poland_2018
1440_Poland_2020
1305_Poland_2020
1482_Poland_2020
MN887188_Poland_2019
708_Poland_2018
586_Poland_2018
516_Poland_2018
MN887186_Poland_2018
483_Romania_2019
1036_Poland_2019
682_Poland_2018
1323_Poland_2020
MN887192_Poland_2018
166_Poland_2017
EU780077_Israel_2006
1950
1960
1970
1980
1990
2000
2010
2020

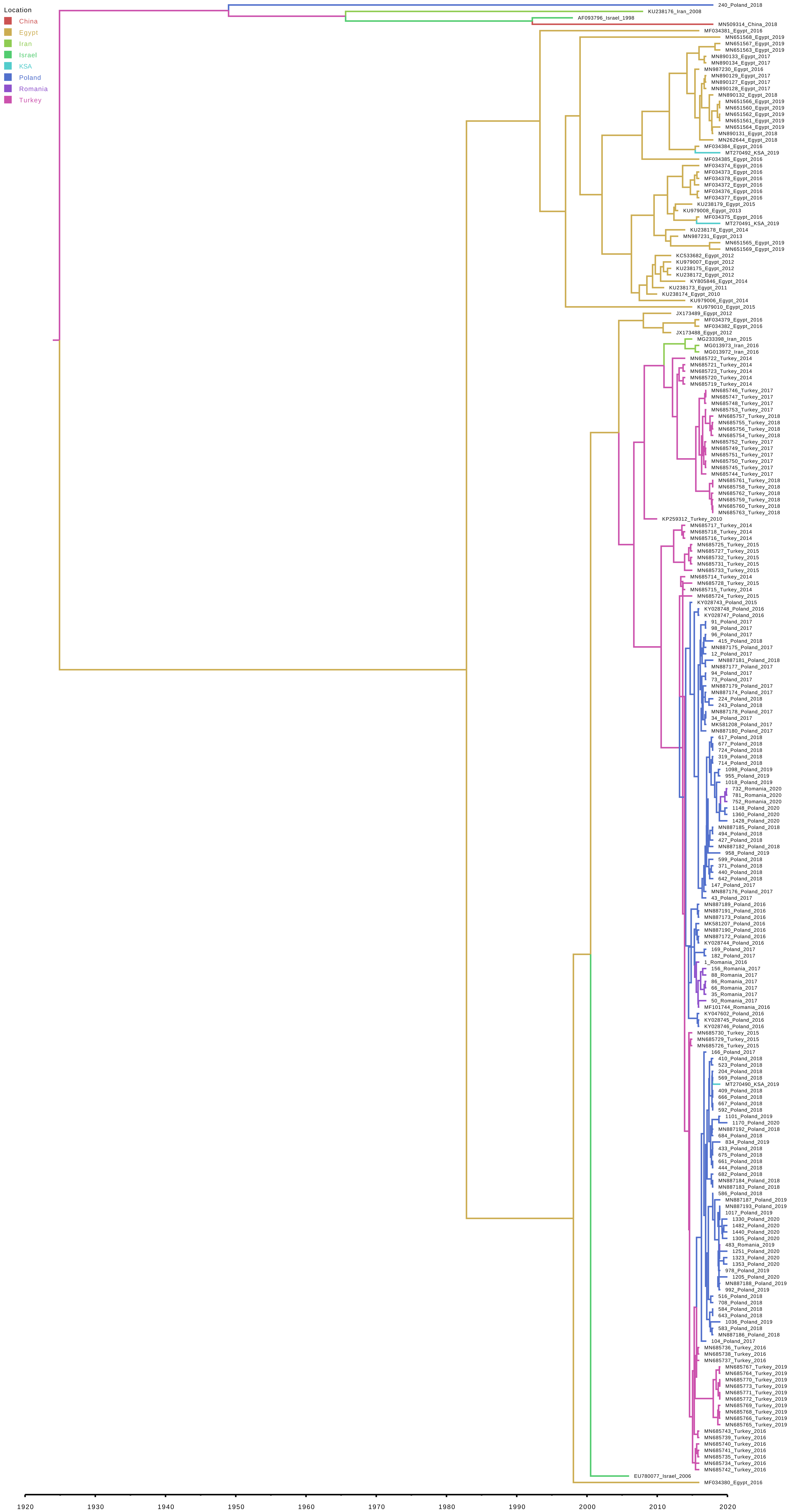

Location
China
Egypt
Iran
Israel
KSA
Poland
Romania
Turkey
240_Poland_2018
KU238176_Iran_2008
AF093796_Israel_1998
MN509314_China_2018
MF034381_Egypt_2016
MN651568_Egypt_2019
MN651567_Egypt_2019
MN651563_Egypt_2019
MN890133_Egypt_2017
MN890134_Egypt_2017
MN987230_Egypt_2016
MN890129_Egypt_2017
MN890127_Egypt_2017
MN890128_Egypt_2017
MN890132_Egypt_2018
MN651566_Egypt_2019
MN651560_Egypt_2019
MN651562_Egypt_2019
MN651561_Egypt_2019
MN651564_Egypt_2019
MN890131_Egypt_2018
MN262644_Egypt_2018
MF034384_Egypt_2016
MT270492_KSA_2019
MF034385_Egypt_2016
MF034374_Egypt_2016
MF034373_Egypt_2016
MF034378_Egypt_2016
MF034372_Egypt_2016
MF034376_Egypt_2016
MF034377_Egypt_2016
KU238179_Egypt_2015
KU979008_Egypt_2013
MF034375_Egypt_2016
MT270491_KSA_2019
KU238178_Egypt_2014
MN987231_Egypt_2013
MN651565_Egypt_2019
MN651569_Egypt_2019
KC533682_Egypt_2012
KU979007_Egypt_2012
KU238175_Egypt_2012
KU238172_Egypt_2012
KY805846_Egypt_2014
KU238173_Egypt_2011
KU238174_Egypt_2010
KU979006_Egypt_2014
KU979010_Egypt_2015
JX173489_Egypt_2012
MF034379_Egypt_2016
MF034382_Egypt_2016
JX173488_Egypt_2012
MG233398_Iran_2015
MG013973_Iran_2016
MG013972_Iran_2016
MN685722_Turkey_2014
MN685721_Turkey_2014
MN685723_Turkey_2014
MN685720_Turkey_2014
MN685719_Turkey_2014
MN685746_Turkey_2017
MN685747_Turkey_2017
MN685748_Turkey_2017
MN685753_Turkey_2017
MN685757_Turkey_2018
MN685755_Turkey_2018
MN685756_Turkey_2018
MN685754_Turkey_2018
MN685752_Turkey_2017
MN685749_Turkey_2017
MN685751_Turkey_2017
MN685750_Turkey_2017
MN685745_Turkey_2017
MN685744_Turkey_2017
MN685761_Turkey_2018
MN685758_Turkey_2018
MN685762_Turkey_2018
MN685759_Turkey_2018
MN685760_Turkey_2018
MN685763_Turkey_2018
KP259312_Turkey_2010
MN685717_Turkey_2014
MN685718_Turkey_2014
MN685716_Turkey_2014
MN685725_Turkey_2015
MN685727_Turkey_2015
MN685732_Turkey_2015
MN685731_Turkey_2015
MN685733_Turkey_2015
MN685714_Turkey_2014
MN685728_Turkey_2015
MN685715_Turkey_2014
MN685724_Turkey_2015
KY028743_Poland_2015
KY028748_Poland_2016
KY028747_Poland_2016
91_Poland_2017
98_Poland_2017
96_Poland_2017
415_Poland_2018
MN887175_Poland_2017
12_Poland_2017
MN887181_Poland_2018
MN887177_Poland_2017
94_Poland_2017
73_Poland_2017
MN887179_Poland_2017
MN887174_Poland_2017
224_Poland_2018
243_Poland_2018
MN887178_Poland_2017
34_Poland_2017
MK581208_Poland_2017
MN887180_Poland_2017
617_Poland_2018
677_Poland_2018
724_Poland_2018
319_Poland_2018
714_Poland_2018
1098_Poland_2019
955_Poland_2019
1018_Poland_2019
732_Romania_2020
781_Romania_2020
752_Romania_2020
1148_Poland_2020
1360_Poland_2020
1428_Poland_2020
MN887185_Poland_2018
494_Poland_2018
427_Poland_2018
MN887182_Poland_2018
958_Poland_2019
599_Poland_2018
371_Poland_2018
440_Poland_2018
642_Poland_2018
147_Poland_2017
MN887176_Poland_2017
43_Poland_2017
MN887189_Poland_2016
MN887191_Poland_2016
MN887173_Poland_2016
MK581207_Poland_2016
MN887190_Poland_2016
MN887172_Poland_2016
KY028744_Poland_2016
169_Poland_2017
182_Poland_2017
1_Romania_2016
156_Romania_2017
88_Romania_2017
86_Romania_2017
66_Romania_2017
35_Romania_2017
50_Romania_2017
MF101744_Romania_2016
KY047602_Poland_2016
KY028745_Poland_2016
KY028746_Poland_2016
MN685730_Turkey_2015
MN685729_Turkey_2015
MN685726_Turkey_2015
166_Poland_2017
410_Poland_2018
523_Poland_2018
204_Poland_2018
569_Poland_2018
MT270490_KSA_2019
409_Poland_2018
666_Poland_2018
667_Poland_2018
592_Poland_2018
1101_Poland_2019
1170_Poland_2020
MN887192_Poland_2018
684_Poland_2018
834_Poland_2019
433_Poland_2018
675_Poland_2018
661_Poland_2018
444_Poland_2018
682_Poland_2018
MN887184_Poland_2018
MN887183_Poland_2018
586_Poland_2018
MN887187_Poland_2019
MN887193_Poland_2019
1017_Poland_2019
1330_Poland_2020
1482_Poland_2020
1440_Poland_2020
1305_Poland_2020
483_Romania_2019
1251_Poland_2020
1323_Poland_2020
1353_Poland_2020
978_Poland_2019
1205_Poland_2020
MN887188_Poland_2019
992_Poland_2019
516_Poland_2018
708_Poland_2018
584_Poland_2018
643_Poland_2018
1036_Poland_2019
583_Poland_2018
MN887186_Poland_2018
104_Poland_2017
MN685736_Turkey_2016
MN685738_Turkey_2016
MN685737_Turkey_2016
MN685767_Turkey_2019
MN685764_Turkey_2019
MN685770_Turkey_2019
MN685773_Turkey_2019
MN685771_Turkey_2019
MN685772_Turkey_2019
MN685769_Turkey_2019
MN685768_Turkey_2019
MN685766_Turkey_2019
MN685765_Turkey_2019
MN685743_Turkey_2016
MN685739_Turkey_2016
MN685740_Turkey_2016
MN685741_Turkey_2016
MN685735_Turkey_2016
MN685734_Turkey_2016
MN685742_Turkey_2016
EU780077_Israel_2006
MF034380_Egypt_2016
1920
1930
1940
1950
1960
1970
1980
1990
2000
2010
2020

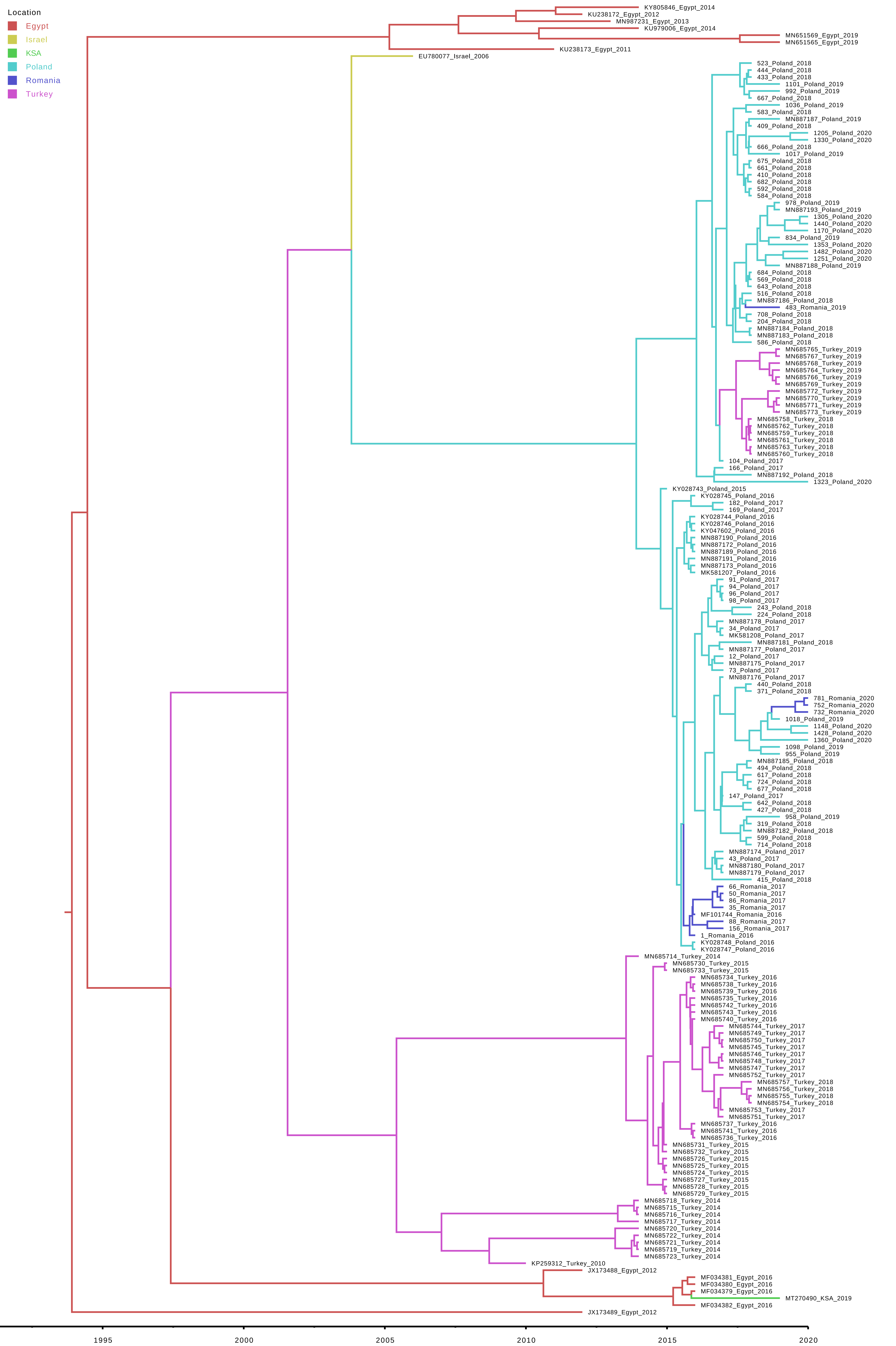

Location
Egypt
Israel
KSA
Poland
Romania
Turkey
KY805846_Egypt_2014
KU238172_Egypt_2012
MN987231_Egypt_2013
KU979006_Egypt_2014
MN651569_Egypt_2019
MN651565_Egypt_2019
KU238173_Egypt_2011
EU780077_Israel_2006
523_Poland_2018
444_Poland_2018
433_Poland_2018
1101_Poland_2019
992_Poland_2019
667_Poland_2018
1036_Poland_2019
583_Poland_2018
MN887187_Poland_2019
409_Poland_2018
1205_Poland_2020
1330_Poland_2020
666_Poland_2018
1017_Poland_2019
675_Poland_2018
661_Poland_2018
410_Poland_2018
682_Poland_2018
592_Poland_2018
584_Poland_2018
978_Poland_2019
MN887193_Poland_2019
1305_Poland_2020
1440_Poland_2020
1170_Poland_2020
834_Poland_2019
1353_Poland_2020
1482_Poland_2020
1251_Poland_2020
MN887188_Poland_2019
684_Poland_2018
569_Poland_2018
643_Poland_2018
516_Poland_2018
MN887186_Poland_2018
483_Romania_2019
708_Poland_2018
204_Poland_2018
MN887184_Poland_2018
MN887183_Poland_2018
586_Poland_2018
MN685765_Turkey_2019
MN685767_Turkey_2019
MN685768_Turkey_2019
MN685764_Turkey_2019
MN685766_Turkey_2019
MN685769_Turkey_2019
MN685772_Turkey_2019
MN685770_Turkey_2019
MN685771_Turkey_2019
MN685773_Turkey_2019
MN685758_Turkey_2018
MN685762_Turkey_2018
MN685759_Turkey_2018
MN685761_Turkey_2018
MN685763_Turkey_2018
MN685760_Turkey_2018
104_Poland_2017
166_Poland_2017
MN887192_Poland_2018
1323_Poland_2020
KY028743_Poland_2015
KY028745_Poland_2016
182_Poland_2017
169_Poland_2017
KY028744_Poland_2016
KY028746_Poland_2016
KY047602_Poland_2016
MN887190_Poland_2016
MN887172_Poland_2016
MN887189_Poland_2016
MN887191_Poland_2016
MN887173_Poland_2016
MK581207_Poland_2016
91_Poland_2017
94_Poland_2017
96_Poland_2017
98_Poland_2017
243_Poland_2018
224_Poland_2018
MN887178_Poland_2017
34_Poland_2017
MK581208_Poland_2017
MN887181_Poland_2018
MN887177_Poland_2017
12_Poland_2017
MN887175_Poland_2017
73_Poland_2017
MN887176_Poland_2017
440_Poland_2018
371_Poland_2018
781_Romania_2020
752_Romania_2020
732_Romania_2020
1018_Poland_2019
1148_Poland_2020
1428_Poland_2020
1360_Poland_2020
1098_Poland_2019
955_Poland_2019
MN887185_Poland_2018
494_Poland_2018
617_Poland_2018
724_Poland_2018
677_Poland_2018
147_Poland_2017
642_Poland_2018
427_Poland_2018
958_Poland_2019
319_Poland_2018
MN887182_Poland_2018
599_Poland_2018
714_Poland_2018
MN887174_Poland_2017
43_Poland_2017
MN887180_Poland_2017
MN887179_Poland_2017
415_Poland_2018
66_Romania_2017
50_Romania_2017
86_Romania_2017
35_Romania_2017
MF101744_Romania_2016
88_Romania_2017
156_Romania_2017
1_Romania_2016
KY028748_Poland_2016
KY028747_Poland_2016
MN685714_Turkey_2014
MN685730_Turkey_2015
MN685733_Turkey_2015
MN685734_Turkey_2016
MN685738_Turkey_2016
MN685739_Turkey_2016
MN685735_Turkey_2016
MN685742_Turkey_2016
MN685743_Turkey_2016
MN685740_Turkey_2016
MN685744_Turkey_2017
MN685749_Turkey_2017
MN685750_Turkey_2017
MN685745_Turkey_2017
MN685746_Turkey_2017
MN685748_Turkey_2017
MN685747_Turkey_2017
MN685752_Turkey_2017
MN685757_Turkey_2018
MN685756_Turkey_2018
MN685755_Turkey_2018
MN685754_Turkey_2018
MN685753_Turkey_2017
MN685751_Turkey_2017
MN685737_Turkey_2016
MN685741_Turkey_2016
MN685736_Turkey_2016
MN685731_Turkey_2015
MN685732_Turkey_2015
MN685726_Turkey_2015
MN685725_Turkey_2015
MN685724_Turkey_2015
MN685727_Turkey_2015
MN685728_Turkey_2015
MN685729_Turkey_2015
MN685718_Turkey_2014
MN685715_Turkey_2014
MN685716_Turkey_2014
MN685717_Turkey_2014
MN685720_Turkey_2014
MN685722_Turkey_2014
MN685721_Turkey_2014
MN685719_Turkey_2014
MN685723_Turkey_2014
KP259312_Turkey_2010
JX173488_Egypt_2012
MF034381_Egypt_2016
MF034380_Egypt_2016
MF034379_Egypt_2016
MT270490_KSA_2019
MF034382_Egypt_2016
JX173489_Egypt_2012
1995
2000
2005
2010
2015
2020

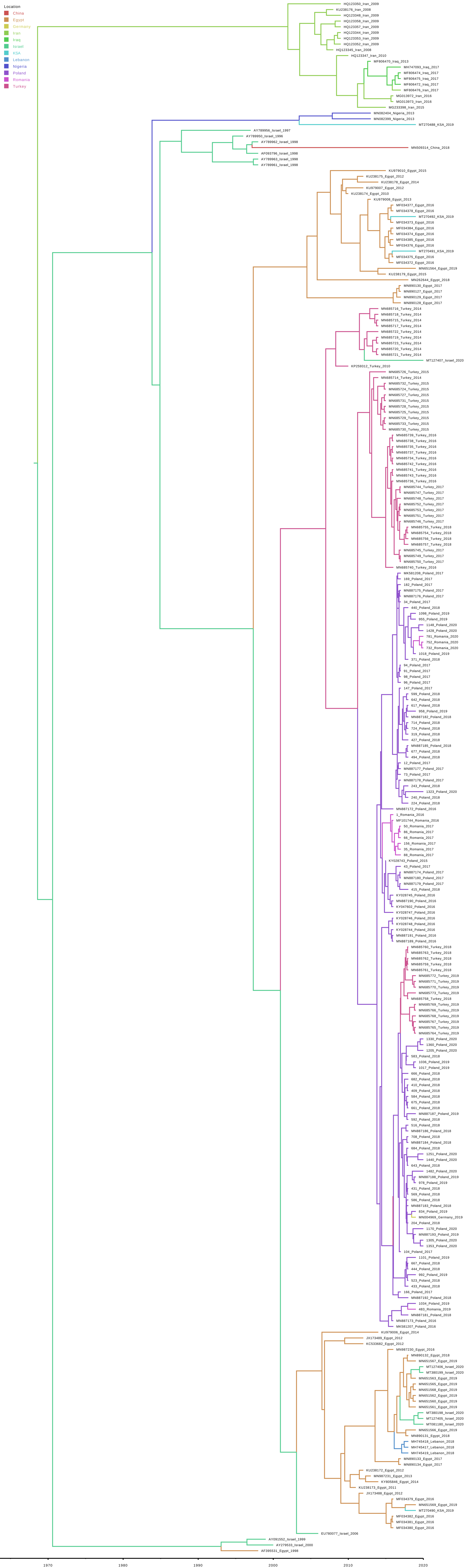
Location
China
Egypt
Germany
Iran
Iraq
Israel
KSA
Lebanon
Nigeria
Poland
Romania
Turkey
1970
1980
1990
2000
2010
2020

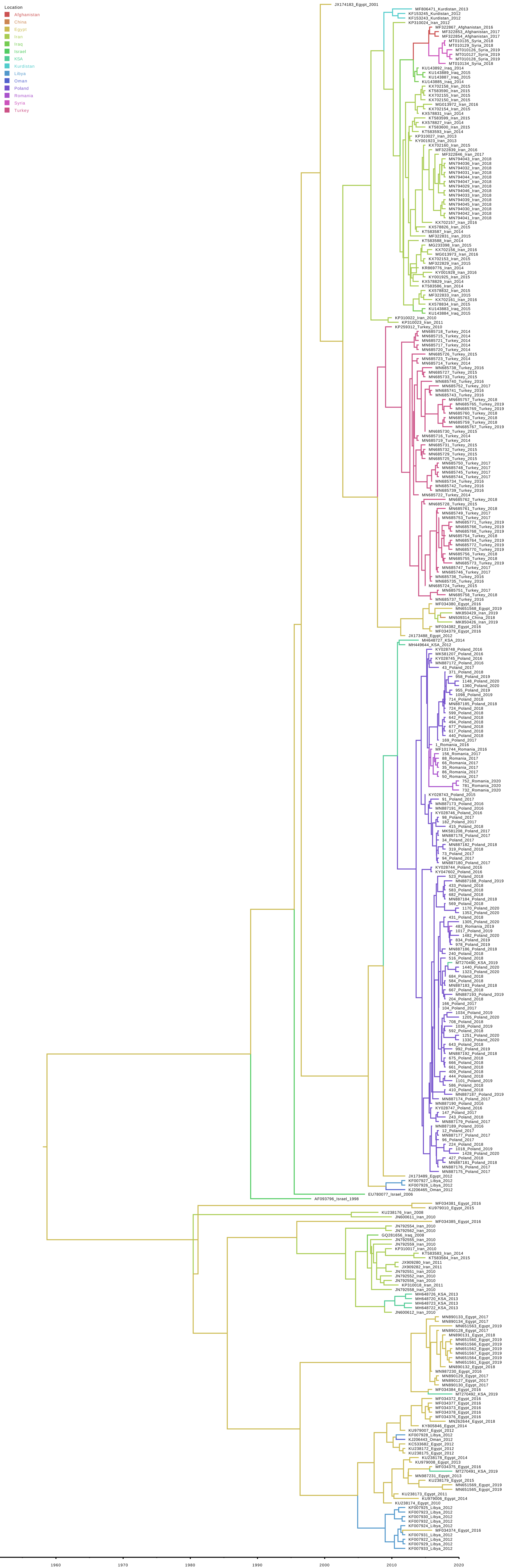
Location
Afghanistan
China
Egypt
Iran
Iraq
Israel
KSA
Kurdistan
Libya
Oman
Poland
Romania
Syria
Turkey
1960
1970
1980
1990
2000
2010
2020
